# Iterative usability testing of a digital joint protection program for people with hand osteoarthritis

**DOI:** 10.1371/journal.pone.0342571

**Published:** 2026-04-09

**Authors:** Dimitra V. Pouliopoulou, Victoria D’Alessandro, Nicole Billias, Joy C. MacDermid, Yuxin (Monica) Lin, Emily Lalone, Ruby Grewal, Pavlos Bobos

**Affiliations:** 1 School of Physical Therapy, Health and Rehabilitation Sciences, Western University, London, Ontario, Canada; 2 Roth McFarlane Hand and Upper Limb Centre, St Joseph’s Healthcare Centre, London, Ontario, Canada; 3 Western’s Bone and Joint Institute, Collaborative Musculoskeletal Health Research Program, London, Ontario, Canada; 4 Department of Surgery, Schulich School of Medicine & Dentistry, Western University, London, Ontario, Canada; 5 Faculty of Health Sciences, Western University, London, Ontario, Canada; 6 Department of Mechanical and Materials Engineering, Western University, London, Ontario, Canada; 7 Epidemiology and Biostatistics, Schulich School of Medicine and Dentistry, Western University, London, Ontario, Canada; Universiteit Twente, NETHERLANDS, KINGDOM OF THE

## Abstract

Hand osteoarthritis is a leading cause of pain, disability, and reduced quality of life in older adults. Joint protection programs are recommended as a core component of self-management, but traditional delivery is limited by barriers to access. Digital programs can overcome these challenges for some people, but their reach and effectiveness depend on usability. We conducted a mixed methods usability study of a remotely delivered joint protection program designed for people with hand osteoarthritis. Twenty-three participants took part, recruited through purposeful sampling to ensure inclusion of groups often underrepresented in research. Usability was assessed using predefined task completion, browser-based eye-tracking, participant ratings, and think-aloud protocols, with iterative refinements applied between participants. Routine navigation tasks, such as navigating between different modules, accessing interactive activities, and viewing short videos, were consistently completed with high success. More complex interactive tasks, including drag-and-drop activities, scenario-based modules, and toggling videos to full screen, initially posed challenges. Over successive iterations, however, usability improved markedly, with later participants achieving near-perfect performance. Qualitative analysis revealed that participants valued clear language, short and focused videos, interactive elements, and the ability to proceed at their own pace, while raising concerns about excessive clicking, unclear instructions, and variation in age representation. Iterative refinements, including platform adjustments, clearer instructions and an introductory video, addressed these issues and contributed to improved performance. This study demonstrates that a remotely delivered, technology-enabled joint protection program for hand osteoarthritis is usable, accessible, and engaging across a diverse sample. Beyond refining the program itself, the study introduces a practical framework for iterative, equity-informed usability testing that can inform the design of future digital health interventions.

## Introduction

Hand osteoarthritis (HOA) is one of the most prevalent musculoskeletal conditions worldwide and is a leading cause of pain, disability, and reduced quality of life in older adults [1; 2]. Globally, HOA is the second most common form of osteoarthritis after knee osteoarthritis and is estimated to affect approximately 142 million people worldwide, with a higher prevalence among women and older adults [1,3,4]. Although radiographic HOA is more common than symptomatic disease, both forms are highly prevalent among adults aged 65 years and older in North America and Europe, affecting up to 60% of this population [5, 2]. In Canada, symptomatic HOA affects approximately one-third of adults aged 65 years and older, underscoring its substantial population-level burden [6].

Joint protection programs (JPPs), are self-management strategies designed for patients with hand arthritis to preserve joint function, reduce pain, and delay symptom progression [7; 1]. JPPs interweave four principal domains: education and self-monitoring, ergonomic retraining, pacing and activity planning, and the prescription of adaptive equipment and environmental modifications [8]. Education provides the foundation, explaining disease mechanisms, identifying high-risk tasks, and encouraging reflection on daily activity patterns [8], while also incorporating behavioural strategies that support sustainable change, such as goal setting, self-monitoring, problem-solving, and building self-efficacy to adopt and maintain joint protection techniques in everyday life [9]. Ergonomic retraining teaches alternative movement techniques, such as avoiding extreme joint postures, using smoother motions, or optimizing handle grip geometry [10]. Pacing strategies involve dividing tasks, interspersing rest breaks, and prioritizing essential duties [8, 11]. Finally, assistive devices, such as built-up handles, electric tools, hands-free technologies, jar openers, and lever taps are prescribed to reduce strain on vulnerable joints [10]. However, traditional delivery of JPPs often occur in in-person rehabilitation settings, which can be difficult to access due to lack of awareness, geographic, mobility, financial, or scheduling barriers [12, 13]. Evidence consistently shows that income, rurality, and transportation barriers restrict access to rehabilitation services, disproportionately affecting individuals most in need [14]. These challenges limit patient engagement and exacerbate inequities in care delivery.

Remotely-delivered interventions provide an opportunity to overcome accessibility barriers by offering flexible, scalable, and patient-centered access to evidence-based strategies [15]. However, evidence from usability evaluations of digital self-management and patient education programs shows that accessibility and effectiveness depend critically on usability, particularly the clarity of content, ease of navigation, and the extent to which users can complete core tasks independently [15]. Previous research on people with HOA shows that if a digital intervention is not intuitive to navigate or its content is difficult to understand, patients are unlikely to engage meaningfully, regardless of the clinical value of the material [12; 13]. Usability testing is therefore an essential step in the development of digital health interventions to ensure accessibility, acceptability, and long-term adoption.

Despite the recognized importance of usability evaluation, many digital health interventions rely on single-round testing or retrospective satisfaction surveys [15]. In contrast, a recent systematic review of digital interventions demonstrates that iterative usability approaches that combine real-time user testing, task-based evaluation, think-aloud methods, and direct observation of user interaction produce tangible improvements in usability and acceptability by aligning intervention design with users’ functional, cognitive, and contextual needs [16]. The review further highlights that early iterative-testing-based refinements can reduce navigation burden, improve comprehension, and increase user engagement, while also supporting cultural sensitivity through clearer language and more relevant content [16]. Such approaches are particularly important for older adults with HOA, who may face unique challenges with digital literacy, visual scanning, or task sequencing.

In addition to methodological limitations, clinical practice– and policy-informed guidance has highlighted the limited consideration of equity within digital health usability research. Guidance on gender-equitable digital health transformation indicates that sex, gender, and other intersecting social and contextual factors shape how digital interventions are accessed, interpreted, and used in practice; when these dimensions are not explicitly considered in usability evaluations, important differences in user experience across populations can remain unrecognized, narrowing the relevance of usability findings, [17,18].

The purpose of this study was to evaluate the usability of a remotely delivered, technology-enabled JPP for people with HOA. We assessed usability through a combination of task completion metrics, error counts, participant ratings, think-aloud protocols, and eye-tracking during navigation with attention to equity-relevant design considerations. We employed an iterative, real-world testing approach in which each participant tested the most current version of the program, and refinements were implemented before subsequent testing. By documenting this process, the study not only identifies specific usability issues and program refinements but also introduces a methodological framework for iterative usability testing that can be applied to the development of other digital health interventions.

## Methods

### Study design

This study was designed as a mixed methods usability evaluation of a newly developed, remotely delivered, technology-enabled JPP for individuals with HOA. The objective was not only to assess usability but also to establish and document a methodological framework for iterative usability testing in digital health interventions. The study integrated eye-tracking, think-aloud protocols, task performance measures, a health literacy assessment, and real-world patient feedback into a continuous cycle of testing and refinement. By structuring the evaluation as a series of iterative cycles, in which each participant engaged with the most current version of the program and their feedback directly informed subsequent refinements, the study aimed to produce practical evidence of usability and a comprehensive methodological framework that could be replicated in other digital health contexts.

### Participants and Recruitment

Eligible participants were adults aged 45 years or older with a clinical diagnosis of HOA and self-reported pain persisting for at least three months. Recruitment took place prospectively between 4 July 2025 and 31 July 2025. Participants were identified from a prior study conducted by our study group [19], in which participants had indicated willingness to be contacted for follow-up research. Patients were approached by the student investigator (DP) using their preferred contact method, either email or telephone. Written informed consent was obtained from all participants prior to any study procedures. No minors were enrolled; therefore, parental/guardian consent was not applicable.

A purposeful sampling strategy was employed to ensure that the sample reflected diversity across sex, gender, socioeconomic status, and other intersectional factors. This approach was central to the methodological framework, as it ensured that usability testing captured the variability of experiences and barriers that different patient subgroups face in engaging with digital health programs. Sex and gender differences are particularly relevant in HOA, as biological and social factors can influence prevalence, disability, and access to care. Other intersectional considerations, such as poverty, rurality, and immigration status, were also considered to strengthen the equity lens of the study.

### Setting

All sessions were conducted remotely through Microsoft Teams to reflect real-world conditions of digital health delivery and reduce accessibility barriers that might arise if the participants were required to travel to our site. Participants could join from their own device or, if they preferred, they were offered the alternative to come in person to the Hand and Upper Limb Centre Clinical Research Lab (HULC-CRL), where they could access the program on a laboratory computer. Participants were asked to share their screens and keep cameras on so that browser-based eye-tracking could be performed using Realeye.io. Microsoft Teams was used to record both audio and screen activity.

### Measures and Equipment

Eye-tracking data were collected using RealEye (RealEye.io), a browser-based webcam eye-tracking platform that enables remote usability testing in naturalistic settings. RealEye estimates gaze position using participants’ built-in webcams and computer vision algorithms. Because this approach relies on consumer-grade hardware, the effective sampling rate is not fixed and varies depending on device specifications, browser performance, and network conditions. In general, webcam-based eye-tracking systems capture gaze data at approximately 30–60 Hz, which is sufficient for identifying fixation patterns, scan paths, and areas of visual attention in usability research, but not for fine-grained oculomotor analyses.

In the present study, eye-tracking data were used to examine fixation locations, scan paths, and skipped content areas during task completion and navigation, consistent with established usability and human–computer interaction research practices [20]. Raw high-frequency gaze metrics were not required, as the focus was on identifying usability barriers and interaction patterns rather than detailed eye movement dynamics.

### Experimental Design

#### Study procedures.

**Fig 1:**
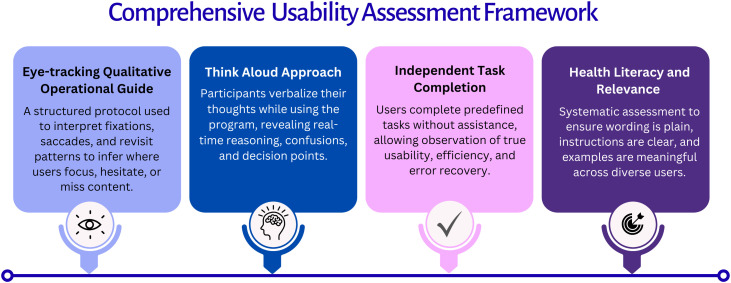
Comprehensive Usability Assessment Framework.

The study followed a structured usability evaluation framework integrating eye tracking, independent task completion, think-aloud protocols, and post-task ratings of content clarity and understanding. This framework was designed to enable concurrent assessment of visual attention, functional task performance, and users’ cognitive and interpretive processes during interaction with a digital health intervention, consistent with established usability and human–computer interaction methodologies [21,20]. An overview of the comprehensive methodological framework is presented in **Fig 1**.

The study employed a between-subjects usability testing design, in which participants were assigned to either a free navigation session or a chunk-specific module testing session. Each participant completed only one session to minimize learning effects and reduce participant burden. Each session focused on a distinct aspect of the program and was structured in two parts.

The first part was identical across both sessions, and incorporated an eye-tracking assessment through a standardized set of pre-defined tasks designed to capture fixation points, scan paths, and skipped areas during navigation. These tasks were administered consistently across participants to assess whether users could successfully complete core functions of the program and to capture errors or difficulties in navigation [21,20]. The same set of pre-defined tasks was used to assess independent task completion. Screen captures illustrating the interface and navigational elements encountered across the pre-defined usability tasks are included in **Appendices S1–S6 in** S1 File.

To standardize exposure and accommodate the technical constraints of webcam-based eye tracking, a fixed time window was applied to the eye-tracking component of each session. Each participant was given 10 minutes to complete the predefined tasks during the eye tracking part of each session. The task set was piloted by the research team a priori to ensure feasibility. The estimated time to complete the 15 tasks as intended was approximately 3 minutes under optimal conditions, making 10 minutes a reasonable limit for capturing task performance under real-world conditions. Participants that run out of time during the eye-tracking session were given the chance to complete the remaining tasks after the end of the eye-tracking session to enable more comprehensive data collection for the independent task completion, that was separate from the eye tracking but used the same task list.

The second part was different for each session. In the free navigation session, participants were encouraged to explore a variety of program content at their own pace. This content included quizzes, videos, drag-and-drop activities, and flip-card exercises. While interacting with the content, they were instructed to verbalize their impressions, challenges, and thought processes in accordance with the think-aloud approach.

In the chunk-specific module testing session, participants were asked to evaluate specific program modules. Each participant engaged with two pre-selected videos chosen by the research team to represent distinct content types, as well as two additional videos of their own choice. After each video, participants were asked to rate the clarity of the language and how easy the information was to understand, including whether they felt confident in explaining it to someone else.

The think-aloud methodology was used to capture participants’ real-time impressions of the program while interacting with it. All participants were guided using a standardized, semi-structured interview guide to ensure consistency across sessions. The interview guides for both sessions are available in **S7 Appendix in** S1 File. Participants were prompted to verbalize what they were doing, what they were trying to achieve, and any difficulties they encountered. They were also encouraged to articulate their reasoning, confusion, and decision-making processes as they navigated or completed tasks. Rather than prompting participants simply to narrate every action, the facilitator encouraged reflection on specific interactions by asking questions such as *“How was that?”* or *“What do you think about that?”* after the completion of a task or while engaging with content. These prompts helped participants articulate their impressions, challenges, and reasoning without interrupting the natural flow of interaction. This approach allowed us to capture both the immediate usability barriers participants encountered and their subjective evaluation of content clarity and navigation.

All participants took part in a final debriefing interview at the end of their session. During this discussion, they were asked to reflect on their overall experience with the program, identify features they found particularly intuitive or confusing, and suggest potential improvements. The debriefing allowed participants to expand on insights raised during the session and ensured that their perspectives were fully captured.

### Technical constraints and adaptations

After data collection had begun, technical constraints were identified that limited eye-tracking assessment for some participants, including incompatibility with Safari browsers and the use of mobile devices, which are not supported by the eye-tracking platform. To avoid excluding participants based on technology access, individuals who were unable to complete eye tracking completed the predefined tasks without eye tracking to allow for the independent task completion assessment and then proceeded to the think-aloud component. Consequently, eye-tracking data were not available for all participants.

### Recruitment, sample size and Iterative Stopping Criterion

Unlike conventional usability studies that pre-specify a fixed number of participants, this study adopted an iterative stopping criterion [22, 23]. Recruitment was guided by two parallel considerations: attainment of thematic saturation for the think-aloud data and acquisition of a sufficient number of eye-tracking datasets. For the think-aloud approach, recruitment proceeded sequentially. Participants were first enrolled into the free navigation session to enable broad exploration of program content and identification of overarching usability issues until thematic saturation was reached, after which recruitment began for the chunk-specific module testing session to inform targeted evaluation of specific content modules. Thematic saturation for the think aloud approach was reached when no new themes, codes, or concepts emerged, all codes had been well-developed, new instances did not lead to further refinement or addition of codes, and the depth and richness of data had been sufficiently explored [22; 23].

Qualitative eye-tracking research for usability testing typically requires approximately 6–8 participants to identify major usability issues and interaction patterns [20]. Recruitment would have continued had either criterion not been met. In the present study, the targeted number of eye-tracking participants was achieved prior to thematic saturation for the second think-aloud session; consequently, recruitment continued until adequate saturation for the think-aloud analysis was reached.

### Data analysis

#### Eye tracking.

**Fig 2:**
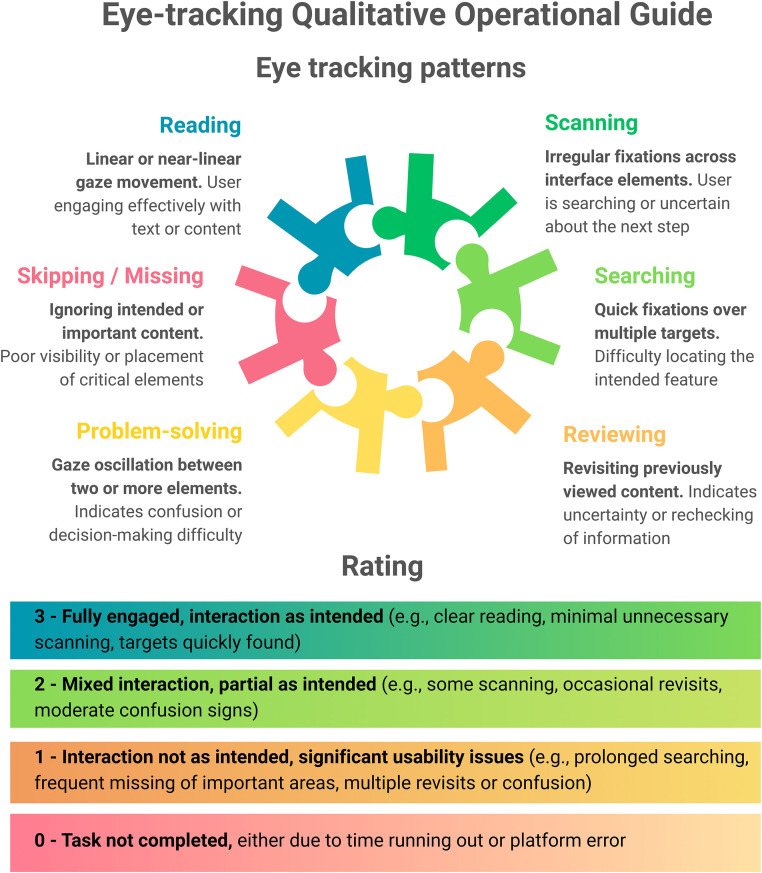
Eye-tracking Qualitative Operational Guide.

Data analysis combined qualitative and quantitative approaches within an iterative feedback loop. Eye-tracking outputs were analyzed independently by two researchers to identify gaze density, inefficient scan paths, and skipped content. The dynamic nature of the platform did not allow for a traditional quantitative eye tracking analysis with state gaze plots and heat-maps [20], hence, the eye tracking data were analysed in a qualitative way [20]. The analysis was guided by a pre-defined operational guide developed in collaboration with a patient interface specialist experienced in usability design and was based on 14 [20] guidelines on eye-tracking for usability studies (**Fig 2**). The framework categorized common gaze behaviours such as reading, scanning, searching, and problem-solving, and provided structured interpretations of what these behaviours indicated about user interaction with the platform. These coded behaviours were mapped onto a four-point rating scale, which generated a single usability score for each participant that captured both navigation intuitiveness and platform-related errors. Each video was reviewed and coded by two independent researchers with experience in eye tracking studies. Disagreements between the two reviewers were resolved through consensus.

Participants were assessed on their ability to complete 15 predefined tasks within a 10-minute period. Each task was rated on the same four-point scale: a score of 3 indicated full engagement and interaction as intended; 2 reflected partial or mixed interaction, where the task was only partly completed as intended; 1 represented interaction that was not as intended and reflected significant usability issues; and 0 was assigned when the task was not completed. A score of 0 could occur either because the participant ran out of time or due to a platform error. Running out of time was interpreted as difficulty navigating the platform efficiently, whereas errors were attributed to technical usability problems. The usability scores for each item were summarized descriptively and visualized over time using descriptive plots to examine patterns of improvement across iterative cycles.

### Independent task completion

Independent task completion was evaluated based on independent completion for all the participants. Tasks completed successfully without assistance were recorded as successful, while instances where participants requested help were recorded as failures to complete. If a task could not be completed due to a platform error, this was noted separately. Independent task completion rates, error counts, and user ratings were summarized descriptively and visualized over time to assess patterns of improvement across iterative cycles.

### Think-aloud

In parallel, transcripts from the think-aloud protocols and debriefing interviews were analyzed thematically using an interpretive descriptive approach tailored to usability research [24,25,26]. All sessions were recorded through Microsoft Teams, transcribed verbatim, and analyzed sentence by sentence through repetitive reading and open coding. Coding was conducted independently by two investigators (DVP and NB). Performing inter-coder reliability suggests the trustworthiness of the coding process, which builds the subsequent analysis [27,28]. The first coder was the researcher who conducted the sessions and brought a clinical perspective to the analysis, while the second coder had a non-clinical background in public health. This combination strengthened the trustworthiness of the analysis by balancing clinical insight with an external lens. Inter-coder agreement was achieved through a constant comparative method, whereby codes were continually compared across and within transcripts, discussed, and refined until consensus was reached [27,28]. Finalized codes were grouped into categories and subsequently developed into higher-order themes that captured recurring usability challenges, barriers to navigation, and areas for refinement [27].

This process was informed by interpretive description, which emphasizes understanding participants’ experiences and contextual meanings [24,25,26]. The lead researcher's clinical background aligned with a constructivist stance that recognized the socially situated nature of participants’ interactions with the program, while the inclusion of a second coder with a non-clinical background contributed a post-positivist orientation that emphasized systematic rigor and minimized bias [27]. These perspectives were integrated pragmatically, ensuring that the analysis produced both a rich, contextually grounded understanding of usability and a transparent, rigorous account of recurring design issues.

### Health literacy, relevance and final de-brief ratings

Participants completed brief rating measures to assess perceived health literacy and overall session difficulty. Perceived health literacy was assessed using a 5-point Likert scale, where higher scores indicated greater ease of understanding of the information presented and greater confidence in explaining the content to others.

At the end of each session, participants also completed a final debriefing difficulty rating using a 0–10 numeric scale, where higher scores indicated greater perceived difficulty. This rating captured participants’ overall experience of interacting with the program, including navigation, task completion, and cognitive effort.

All rating data were summarized descriptively and used to complement eye-tracking, task performance, and qualitative think-aloud findings.

### Data Processing and Figure Generation

Quantitative usability scores, task completion rates, error counts, and participant ratings were aggregated descriptively using Microsoft Excel. Figures presented in the Results section were generated from these aggregated datasets and exported as charts summarizing usability scores, task performance, and changes across iterative cycles. Final figure layout and visual formatting (e.g., labeling, color consistency, and alignment) were completed using Canva to enhance clarity and readability, without altering the underlying data. Figures presented in the Methods section were also created using Canva and illustrate the conceptual and analytical frameworks developed to guide the study design and data analysis. These figures provide a visual representation of key methodological components and their relationships, facilitating interpretation of the procedures described in the text.

### Ethical considerations

The study was reviewed and approved by the Western University Research Ethics Board (Study ID: 126578) prior to the commencement of recruitment and data collection. The ethics review evaluated the study protocol, consent procedures, and data management plan, including the use of remote data collection, browser-based eye-tracking technology, and screen sharing. Participants were provided with a Letter of Information detailing the study purpose, procedures, risks, benefits, and data handling processes, which they were able to review at their own pace prior to participation. Written informed consent was obtained prior to the commencement of any study procedures. Participants were informed that withdrawal was possible at any time without consequence, and that in the event of withdrawal after data collection had begun, they would be asked whether previously collected data could be retained or should be withdrawn from the study.

Data were anonymized, securely stored, and stripped of personal identifiers in accordance with institutional ethics guidelines. The browser-based eye-tracking platform (RealEye.io) did not store video images of participants, capturing only gaze coordinates and mouse behavior. To further protect confidentiality, participants were instructed to close any windows containing personal or identifiable information prior to screen sharing.

## Results

A total of 23 participants were recruited for this study. The demographics of the sample are presented in **Table 1**. Purposeful sampling ensured representation of groups that are often underrepresented in research. Specifically, four participants (17.4%) were immigrants, seven (30.4%) lived in rural communities, and seven (30.4%) reported caring responsibilities for children or adults. Five participants (21.7%) did not have university-level education, and nine (39.1%) identified as living with a disability, including physical/functional (30.4%), sensory (8.7%), mental health-related (8.7%), and cognitive/learning-related conditions (4.3%).

**Table 1:** Population Characteristics.

Variable	Category	n (%)
**Sex**	Female	17 (73.9)
	Male	6 (26.1)
**Gender Identity**	Woman	17 (73.9)
	Man	6 (26.1)
**Marital Status**	Married	13 (56.5)
	Single	3 (13.0)
	Separated, but still legally married	3 (13.0)
	Widowed	2 (8.7)
	Common-law	1 (4.3)
	Divorced	1 (4.3)
**Household Size**	1	7 (30.4)
	2	11 (47.8)
	4	3 (13.0)
	5	1 (4.3)
	6	1 (4.3)
**Caring Responsibilities**	Yes	7 (30.4)
	No	16 (69.6)
**Employment Status**	Retired	17 (73.9)
	Employed	3 (13.0)
	Long-term disability (LTD)	2 (8.7)
	Prefer not to say	1 (4.3)
**Education**	Higher education	18 (78.3)
	Trades	5 (21.7)
**English as First Language**	Yes	20 (87.0)
	No	3 (13.0)
**Immigrants**	No	19 (82.6)
	Yes	4 (17.4)
**Rurality**	No	16 (69.6)
	Yes	7 (30.4)
**Socioeconomic Status**	Can afford daily needs + extra	16 (69.6)
	Can afford daily needs, limited extras	6 (26.1)
	Prefer not to say	1 (4.3)
**Race**	White	18 (78.3)
	European	5 (21.7)
	Asian	2 (8.7)
	South Asian	2 (8.7)
	East Asian	1 (4.3)
	Indigenous (Turtle Island)	1 (4.3)
**Disability**	Yes	9 (39.1)
	No	14 (60.9)
**Disability Type**	Physical/functional	7 (30.4)
	Sensory	2 (8.7)
	Mental health-related	2 (8.7)
	Cognitive/learning	1 (4.3)
	Multiple	2 (8.7)

Eye tracking analysis and independent task completion.

**Fig 3:**
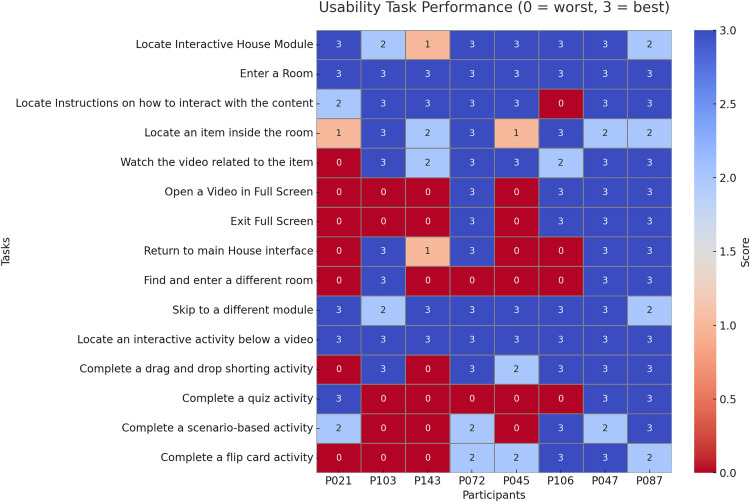
Eye tracking coding score. Higher scores represent interaction as intended.

**Fig 4:**
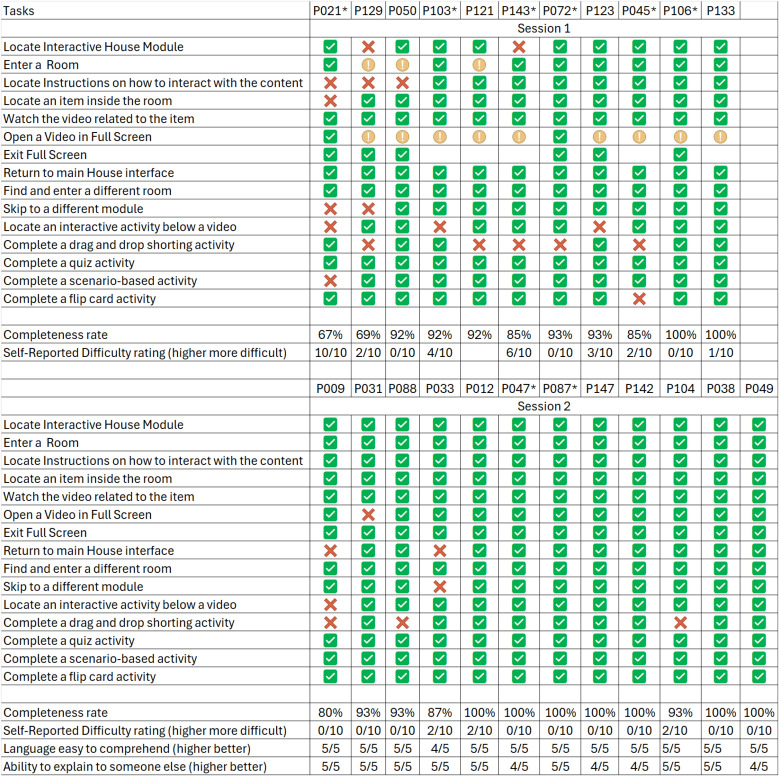
Independent Completion Rate for each participant. Participants marked with * completed the tasks while eye tracking. Check-marks indicate independent completion; X indicates non-independent completion;! indicates platform errors.

Eight participants took part in the eye-tracking analysis. A summary of the eye-tracking coding score is presented in **Fig 3**, with detailed coding results provided in **S8 Appendix in** S1 File. Task completion ratings were obtained from all 23 participants and are summarised in **Fig 4**.

Across tasks, the highest levels of usability were observed for routine navigation actions such as entering a room, locating interactive activities below a video, and watching short videos associated with items. These tasks were consistently rated at or near the maximum score of three, indicating that participants generally found them straightforward and intuitive. Similarly, the skip-to-module function and flip card activities achieved above-average scores, suggesting that once participants understood the navigation structure, moving across content and completing structured exercises was relatively easy.

In contrast, several tasks revealed substantial usability challenges. The most challenging tasks overall were exiting and re-entering different rooms, which often resulted into platform errors, especially in participants with less stable internet connection, highlighting barriers in returning to the main house interface or moving between rooms. Opening and exiting full-screen videos were also consistently problematic, with multiple participants unable to complete the task due to errors. Drag-and-drop sorting activities and quizzes generated mixed results. Although there were no errors in the platform these activities averaged low to moderate scores; some participants could complete them successfully while others struggled with functionality or clarity. Scenario-based activities produced similar variability, reflecting that not all interactive elements were equally intuitive.

Over time, however, these difficulties gradually resolved, as indicated by the continuously improved numbers and the perfect or close to perfect scores in the final participants. Tasks that initially caused hesitation became easier for participants as iterative refinements were made. Later groups were more likely to complete these activities successfully and with less confusion, suggesting that the platform adjustments contributed to smoother performance.

### Qualitative Results.

A total of 3 themes emerged from our qualitative analysis: (1) perceived clarity and comprehension of content; (2) navigation, interactivity, and user experience; and (3) relevance, representation, and suggested improvements. An overview of the themes, including codes and quotes is presented in **Table 2**.

**Table 2:** Thematic analysis.

Theme	Sub-theme	Exemplar Quotes
**1. Clarity and Comprehension of Content**	Clear language & presentation	*“Very easy to understand. It’s very clear, very clear.”* (P142) *“I like that they're short and sweet and directly to the point.”* (P036)
	Tone of voice	*“[Her] voice is more soothing…It’s just easier to listen to.”* (P036) *“The voice and tone are perfect…It’s easy to understand and follow.”* (P087)
		
**2. Navigation, Interactivity, and User Experience**	Intuitive navigation	*“It was very easy to navigate…I had no problem.”* (P038) *“It’s pretty consistent with other platforms.”* (P047)
	Value of interactivity	*“The flip cards…repeat and put it into your mind again, what was explained in the video.”* (P147) *“The interactive house was the finest part of the whole thing.”* (P021)
	Usability concerns	*“The less clicking the better…when my hands are bad, any clicking on the keyboard is painful.”* (P106) *“I would prefer [the video] wouldn't go away.”* (P129)
		
**3. Relevance, Representation, and Suggested Improvements**	Representation & relevance	*“I like that…you've included different activities to apply for different people of different ability levels.”* (P072) *“There were many different potential things…not just the older lady with her gnarled fingers, but then there was a person painting and then the person playing golf.”* (P045)
	Reinforcement vs. new learning	*“It wasn't really new information…but it reinforced what I had already been told.”* (P050) *“I didn't realize how much pressure some small tasks caused on my hands and fingers.”* (P009)
	Perceptions of age-appropriateness	*“Some of the components seemed too juvenile.”* (P045) *“I don't want something that makes me feel like I'm 100 years old…make it younger looking.”* (P087)
	Suggested improvements	*“Maybe add raised toilet seats in the bathroom section.”* (P021) *“Having references or links to arthritis societies and physiotherapy resources would be really helpful.”* (P047) *“If there was a module on ‘arthritis expanding I would come back to it.”* (P087)

*Exemplar participant quotes are provided for each theme and sub-theme to illustrate key aspects of program usability. Quotes have been lightly edited for readability without altering meaning. Participant IDs (e.g., P036) indicate unique study participants.*

### Theme 1: Perceived Clarity and Comprehension of Content

Participants consistently highlighted the clarity of the program's language and visual presentation. Most described the videos as *“very easy to understand”* (P142) and *“short and sweet, directly to the point”* (P036). Ratings of ease of understanding were frequently at the upper end of the 5-point scale, with many participants reporting confidence in explaining the material to others: *“Very confident because…not only do you describe it…visually, but also verbally”* (P104).

Several participants also noted the tone of voice in the narration as an important facilitator of comprehension and engagement, describing it as *“soothing”* (P036) and *“perfect…very clear to understand…it's easy to follow”* (P087). This was seen as contributing to a positive, accessible learning experience.

### Theme 2: Navigation, Interactivity, and User Experience

Ease of navigation emerged as a central theme. Many participants rated the platform highly intuitive: *“It was very easy to navigate…I had no problem”* (P038). Ratings typically clustered between 4–5 out of 5, with some participants describing the interface as consistent with other digital platforms they were familiar with (P047).

Interactive elements such as the “flip cards” and “interactive house” were particularly valued. Participants described the flip cards as *“simple to follow”* (P142) and effective for reinforcing key points: *“It repeats and puts it into your mind again, what was explained in the video”* (P147). The interactive house was highlighted as *“the finest part of the whole thing”* (P021), because it allowed self-paced exploration of different rooms and tasks: *“I like that you can click on different items…so things that would be more like the kitchen were more meaningful for me because I'm in there more”* (P106).

Despite positive feedback, several participants identified usability issues. For instance, the drag-and-drop exercises were not always intuitive: *“I didn't know that was the card…maybe highlight it flashing”* (P009).

Importantly, participants noted that excessive clicking could cause strain for individuals with hand pain. As one participant explained, *“the less clicking the better…when my hands are bad, any clicking on the keyboard is painful”* (P106). Similar frustrations arose with hover-triggered videos that disappeared too quickly, with participants requesting they remain visible: *“I would prefer that it wouldn't go away”* (P129).

The font size was good for the majority of the participants, both when they were using a laptop/desktop and when they were using their mobile devices. Accessibility concerns with regards to the font size of the subtitles were raised from one participant (P021) when the videos are not in a full screen mode, but they were not a concern when the video was on full screen mode. Technological literacy concerns were also raised from some participants, suggesting that scrolling to access content lower in the page was not intuitive to them (P021, P147).

### Theme 3: Relevance, Representation, and Suggested Improvements

Participants emphasized the importance of tailoring content to reflect their own lived experiences. Several reported feeling represented by the variety of tasks and examples included: *“I like that…you've included different activities to apply for different people of different ability levels”* (P072). Others valued the mix of voices and graphics, describing the videos as *“inviting, the whole app is pleasant”* (P047).

For many, the content reinforced prior knowledge of joint protection strategies: *“It wasn't really new information for me in there, but it certainly reinforced what I had already been told”* (P050). Others described discovering new strategies that were directly applicable: *“I didn't realize how much pressure some small tasks caused on my hands and fingers”* (P009), or, *“I never considered using something like [a key holder]”* (P009). Participants appreciated practical and relatable examples, especially when they introduced devices they had not encountered before.

Participants differed in how age-appropriate they found the content. One participant perceived certain features as *“too juvenile”* (P045) while another suggested that the features were geared toward older adults (P087). The rest of the participants saw the content as practical and relevant across age groups.

Participants recommended adding content on pain management, nutrition and exercises. Some asked for an evolving module to reflect disease progression: *“It’s not intuitive for me to come back to it [as my arthritis is progressing]…if there was another module like ‘arthritis is expanding I would”* (P087). Others requested integration of resources such as links to physiotherapy exercises, assistive device suppliers, or arthritis societies: *“Having references…where could I go for help…that's really helpful”* (P047).

Overall, participants reported that the program was easy to navigate, engaging, and clear, with interactive features enhancing usability and motivation. While much of the content reinforced prior knowledge, many participants also learned new strategies they found practical and applicable. Concerns centered on the physical burden of repeated clicking, the lack of clear instructions in some of the interactive activities, and the fact that scrolling did not feel intuitive. Divergent views on age appropriateness indicated that while some participants perceived certain elements as either too juvenile or too focused on older adults, this variation may demonstrate that the program accommodates a wide spectrum of users. These qualitative findings, triangulated with task performance metrics and eye-tracking scores, informed iterative refinements to enhance both accessibility and relevance of the program.

### Overview of Iterative Program Refinements

To improve user experience, one of the first changes addressed the clarity of instructions. Initially, participants had to click on a questionnaire-style icon to access guidance, which added extra steps and made instructions less visible. Replacing the icon with plain text reduced unnecessary clicks and made the instructions easier to locate and follow.

Another early barrier involved use of the sidebar menu to enter different modules. Although the menu itself was easy to find, it included a small button that allowed users to mark modules as complete. Participants with less computer experience frequently clicked on this button instead of the module heading, which prevented the module from opening and caused confusion. To resolve this, the “mark as complete” function was disabled. Both changes, implemented after participant 6 (P143), significantly improved navigation.

To further support participants, we developed an introductory video that provided step-by-step guidance on platform navigation. The video demonstrated how to scroll through content, toggle the sidebar, and engage with interactive features such as drag-and-drop activities. The first version was introduced with participant 13 (P031), and refinements were made after feedback from participants 13–15 (P031, P088, P033). The final version, first tested with participant 16 (P012), led to a marked improvement in usability. Two versions of the video were created, one optimized for desktop and laptop users, and one tailored for mobile devices to reflect interface differences.

Several refinements were also made to address platform errors, particularly within the interactive house feature. Early in testing, larger rooms containing more than 9–10 items often failed to load properly, especially for participants with slower internet connections or those accessing the program on mobile devices. By restructuring the house into two floors, increasing the number of rooms, and reducing the number of items per room, the interface of each room became simpler and more reliable. These modifications resolved all platform-related loading issues by participant 12 (P009).

## Discussion

This study evaluated the usability of a remotely delivered, technology-enabled Joint Protection Program (JPP) for people living with hand osteoarthritis. Using a combination of task-based assessments, eye-tracking, participant ratings, and think-aloud protocols, we identified areas of challenge and refinement while demonstrating that patients were able to successfully navigate the program and interact with diverse content features, including videos, quizzes, and interactive activities. In the early phases of testing, core navigation and short, structured learning tasks were well received, but more complex interactive components such as full-screen toggling, drag-and-drop activities, and cross-room navigation posed significant usability barriers. These challenges reflected both technical barriers in the platform and the natural learning curve as users engaged with unfamiliar features for the first time.

Task completion rates and navigation ratings improved over successive iterations, reflecting the impact of real-time modifications to the program. This progression highlights the value of iterative design in reducing recurring barriers and making the program more usable. Issues that initially hindered interaction were progressively addressed, leading to clearer task completion and more intuitive navigation as testing advanced. Beyond troubleshooting platform errors, one key refinement was the addition of an introductory video that guided participants step by step through platform navigation and explained how to use features that had posed difficulties in the early testing cycles. The findings indicate that the final improved version of the JPP could be delivered entirely online in a way that is both intuitive and accessible to a diverse patient population, addressing a critical gap in musculoskeletal rehabilitation.

Beyond the usability findings, a key contribution of this work is methodological. Unlike most digital intervention studies, which rely on retrospective satisfaction surveys or interviews [16], we employed an iterative, participant-by-participant refinement process that includes structured observation, think-aloud cognitive interviewing, and eye-tracking to triangulate findings, strengthen rigor, and mitigate biases that arise when assessments rely solely on researchers’ interpretations. Each participant engaged with the most current version of the program, and their feedback directly informed improvements that were implemented before subsequent testing. This design allowed usability barriers to be identified and resolved in real time, reducing the risk of late-stage usability failures and enhancing the likelihood that the program will be acceptable in larger-scale trials. Our findings therefore highlight not only the feasibility of the JPP itself but also introduce a reproducible framework for iterative usability testing in digital health interventions.

This methodological framework is particularly relevant for populations such as older adults with hand osteoarthritis, who may face unique barriers related to digital literacy, vision, or navigation. By combining direct observation, performance metrics, and qualitative feedback, the approach captures a fuller picture of usability than any single method alone [18; 20]. It also provides a structured pathway between prototype development and pilot testing, ensuring that interventions are optimized before they are deployed in clinical research.

An additional methodological strength of this study was the integration of an equity lens into the usability evaluation. Purposeful sampling ensured representation across sex, gender, socioeconomic status, and other intersectional factors, reflecting the reality that digital health interventions are not adopted uniformly across populations. Previous research has shown that women, gender-diverse individuals, and those experiencing socioeconomic disadvantage often face disproportionate barriers in accessing and benefiting from rehabilitation services [8]. These disparities may be amplified in digital environments if usability is tested only in narrow, homogeneous populations [15; 18]. By deliberately recruiting a diverse sample and analyzing how usability issues emerged across different subgroups, this study provides a model for equity-informed usability testing. Embedding such an approach into early stages of intervention development is critical for designing programs that are not only functional and engaging but also inclusive and accessible across diverse patient groups.

This study has several strengths. It is one of the first to systematically evaluate the usability of a fully online JPP for HOA, integrating task performance, eye-tracking, and think-aloud protocols

within an iterative refinement process. The methodological design where each participant tested the most updated version of the program and their feedback directly informed subsequent improvements represents a departure from conventional single-round usability studies. This continuous improvement cycle, combined with an equity-focused sampling strategy, enhances both the robustness and the generalizability of our findings.

The methodological framework that was used for the coding of the eye tracking videos offers a standardised methodology for dynamic platforms and can be applied to the development of other digital health interventions. By embedding iterative, equity-informed usability testing early in the design process, digital health developers can create interventions that are more intuitive, accessible, and responsive to the diverse needs of patients.

Nevertheless, some limitations should be acknowledged. The study focused on short-term usability and did not evaluate long-term engagement, adherence, or clinical effectiveness of the intervention. While browser-based eye-tracking provided valuable insights into navigation patterns, its accuracy is lower than that of laboratory-grade systems; however, its feasibility and scalability make it well-suited for real-world usability testing. Longer-term studies are needed to assess sustained engagement and whether usability translates into improved self-management and functional outcomes.

## Conclusion

This study demonstrated that a remotely delivered, technology-enabled Joint Protection Program for people with hand osteoarthritis is usable, accessible, and capable of supporting patient interaction with diverse digital content. Through the integration of task-based assessments, eye-tracking, participant ratings, and think-aloud protocols within an iterative testing framework, we identified and resolved usability challenges in real time, improving navigation and comprehension across successive versions of the program. By embedding an equity lens into sampling and design, this work also highlighted the importance of ensuring that digital health interventions are inclusive and responsive to diverse patient needs. Beyond informing the refinement of the JPP itself, the study introduces a methodological framework for iterative, equity-informed usability testing that can guide the development of future digital health interventions.

## Supporting information

S1 File Appendix. (DOCX)
