# Supplementary material for: Iterative usability testing of a digital joint protection program for people with hand osteoarthritis
Source: PLoS One. 2026 Apr 9;21(4):e0342571. doi: 10.1371/journal.pone.0342571 (PMC13065036; doi:10.1371/journal.pone.0342571)
Supplement: S1 File — (DOCX) [file pone.0342571.s001.docx]

**Title**

Iterative Usability Testing of a Digital Joint Protection Program for People with Hand Osteoarthritis

**Authors**

Dimitra V Pouliopoulou, PT, MSc Victoria D’Alessandro, BHSc Nicole Billias, BPH Joy C MacDermid, PT, PhD Yuxin (Monica) Lin, MBDC Emily Lalone, PhD Ruby Grewal, MD, MSc, FRCSC Pavlos Bobos, PT, PhD

Table of Contents

[**S1 Appendix. Platform navigation bar** 2](#_Toc217987594)

[**S2 Appendix. Interactive house activity – outline** 3](#_Toc217987595)

[**S3 Appendix. Interactive house activity – room example** 4](#_Toc217987596)

[**S4 Appendix. Drug and drop activity** 5](#_Toc217987597)

[**S5 Appendix. Flip card activity** 6](#_Toc217987598)

[**S6 Appendix. Quiz** 7](#_Toc217987599)

[**S7 Appendix. Interview Guides** 8](#_Toc217987600)

[**S8 Appendix. Eye tracking coding** 12](#_Toc217987601)

# **S1 Appendix. Platform navigation bar**


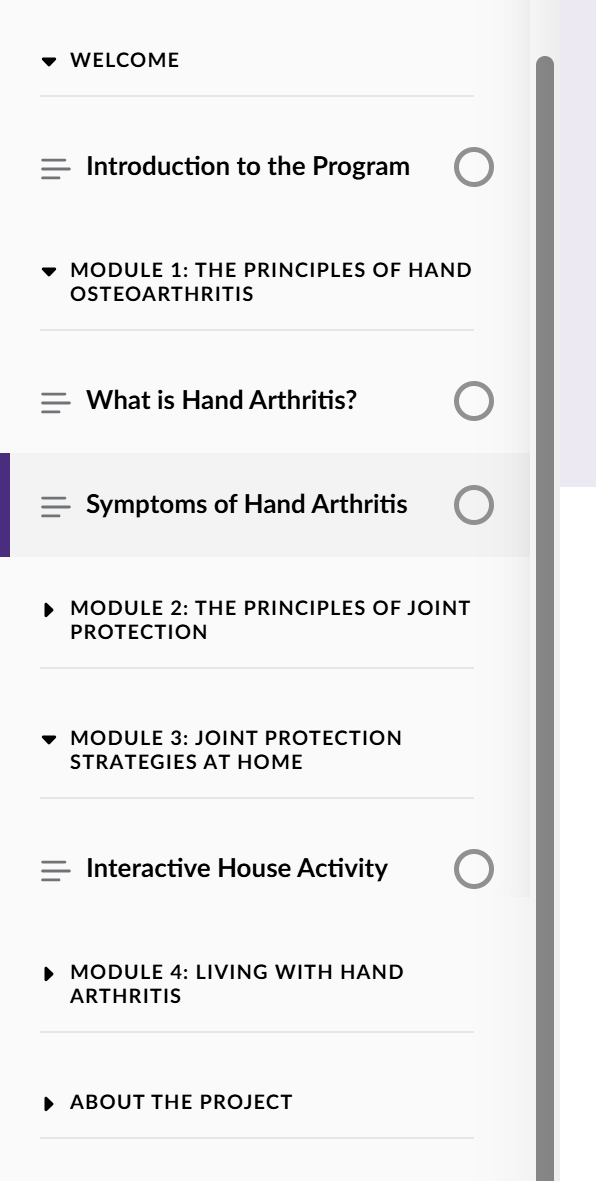


# **S2 Appendix. Interactive house activity – outline**


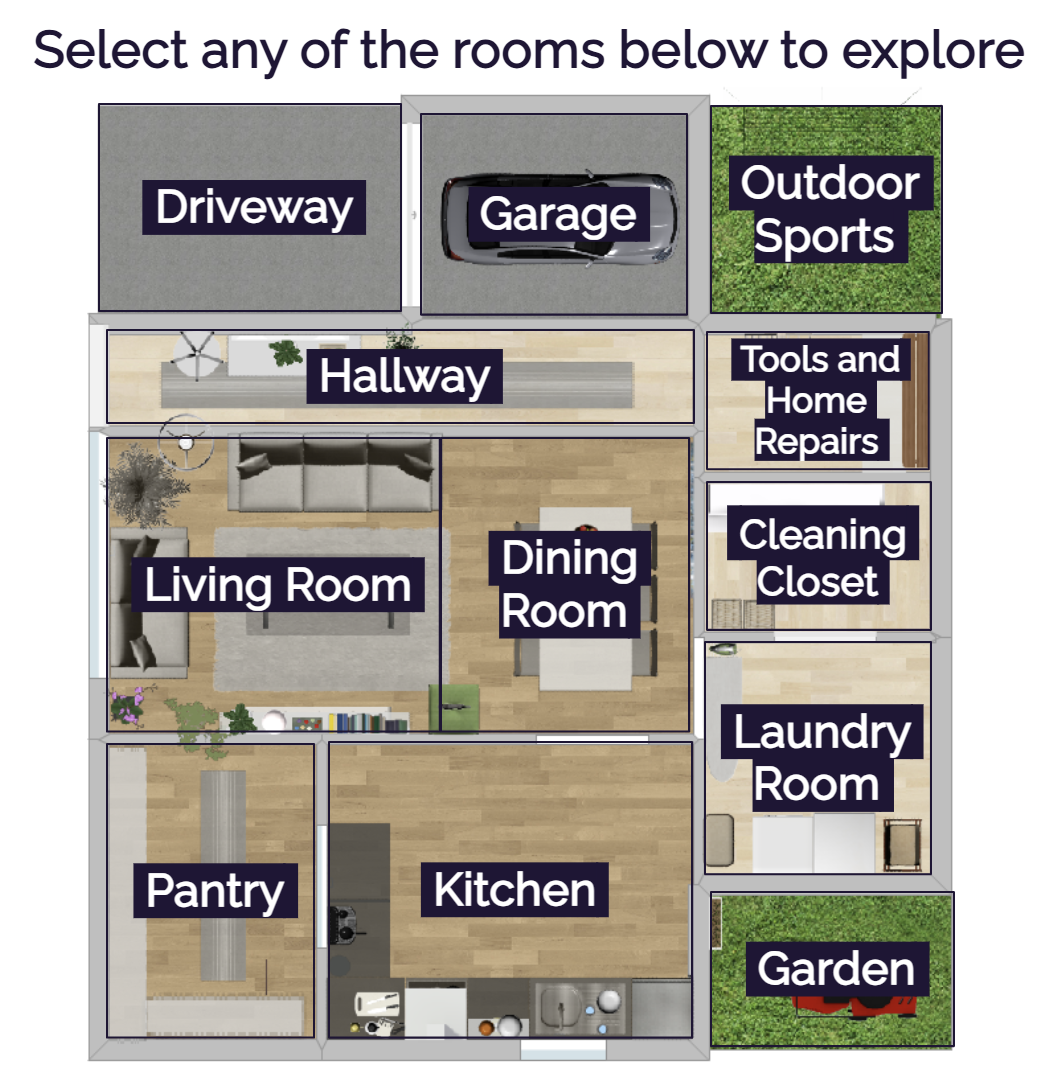


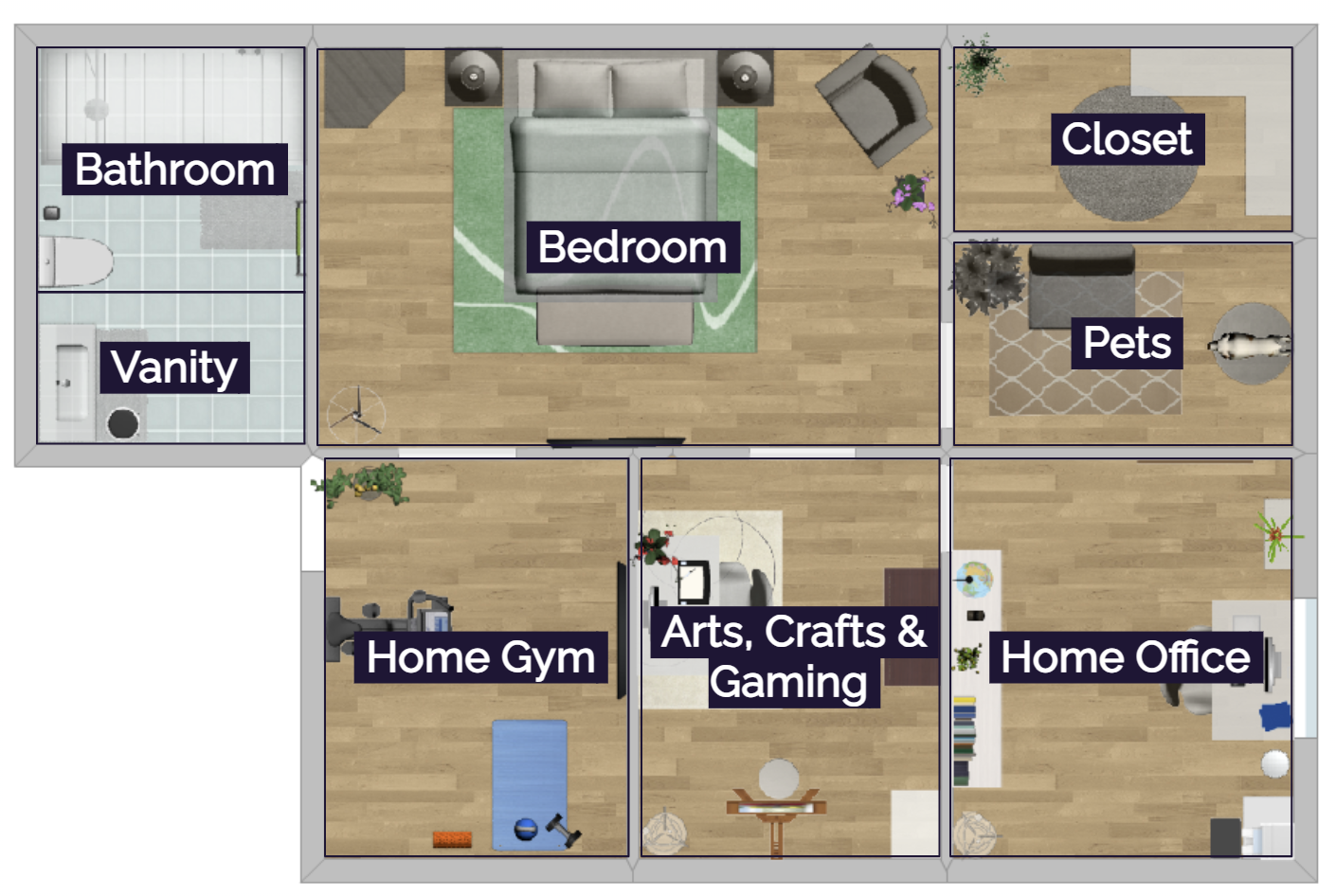


# **S3 Appendix. Interactive house activity – room example**


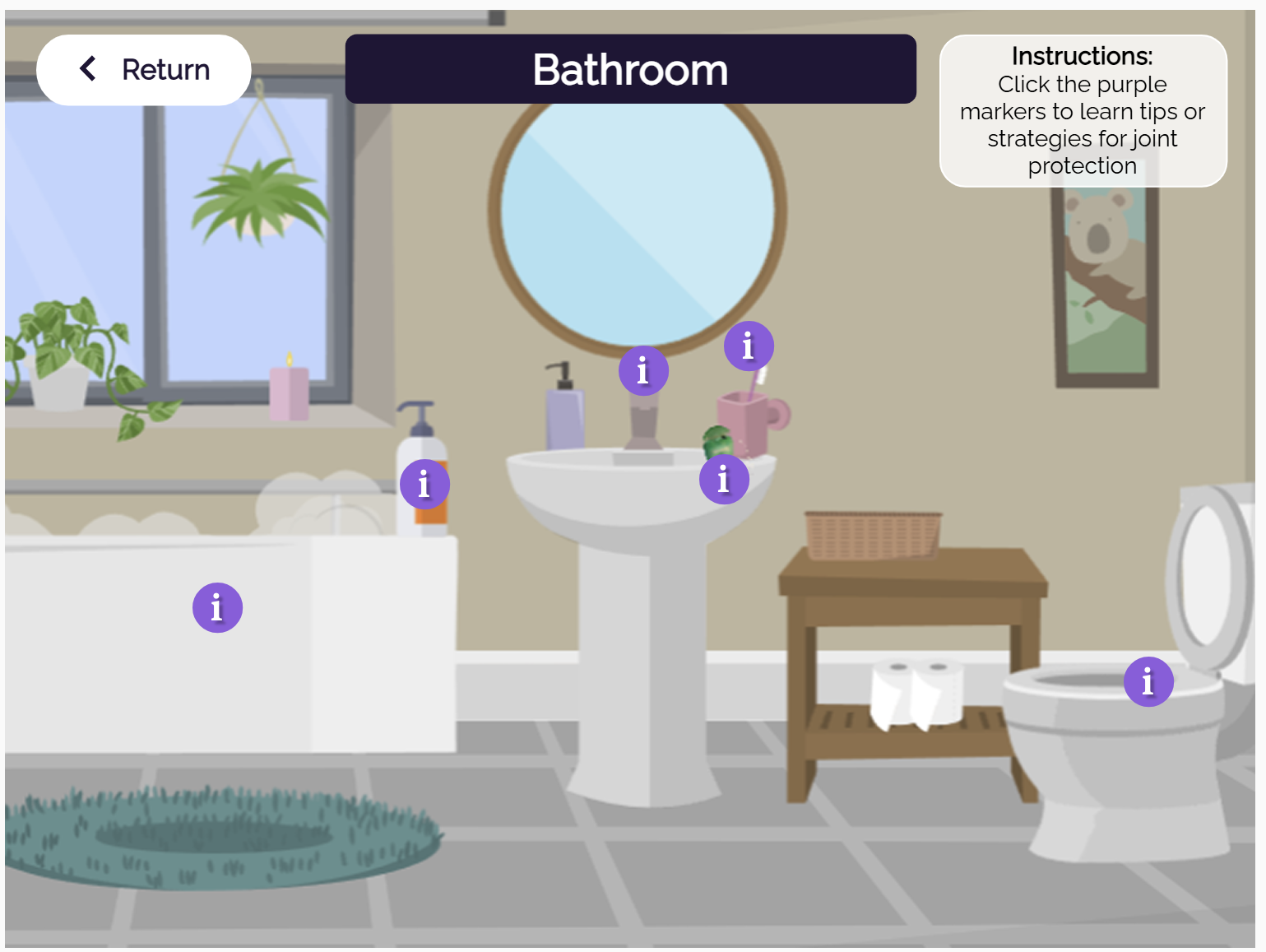


# **S4 Appendix. Drug and drop activity**

**
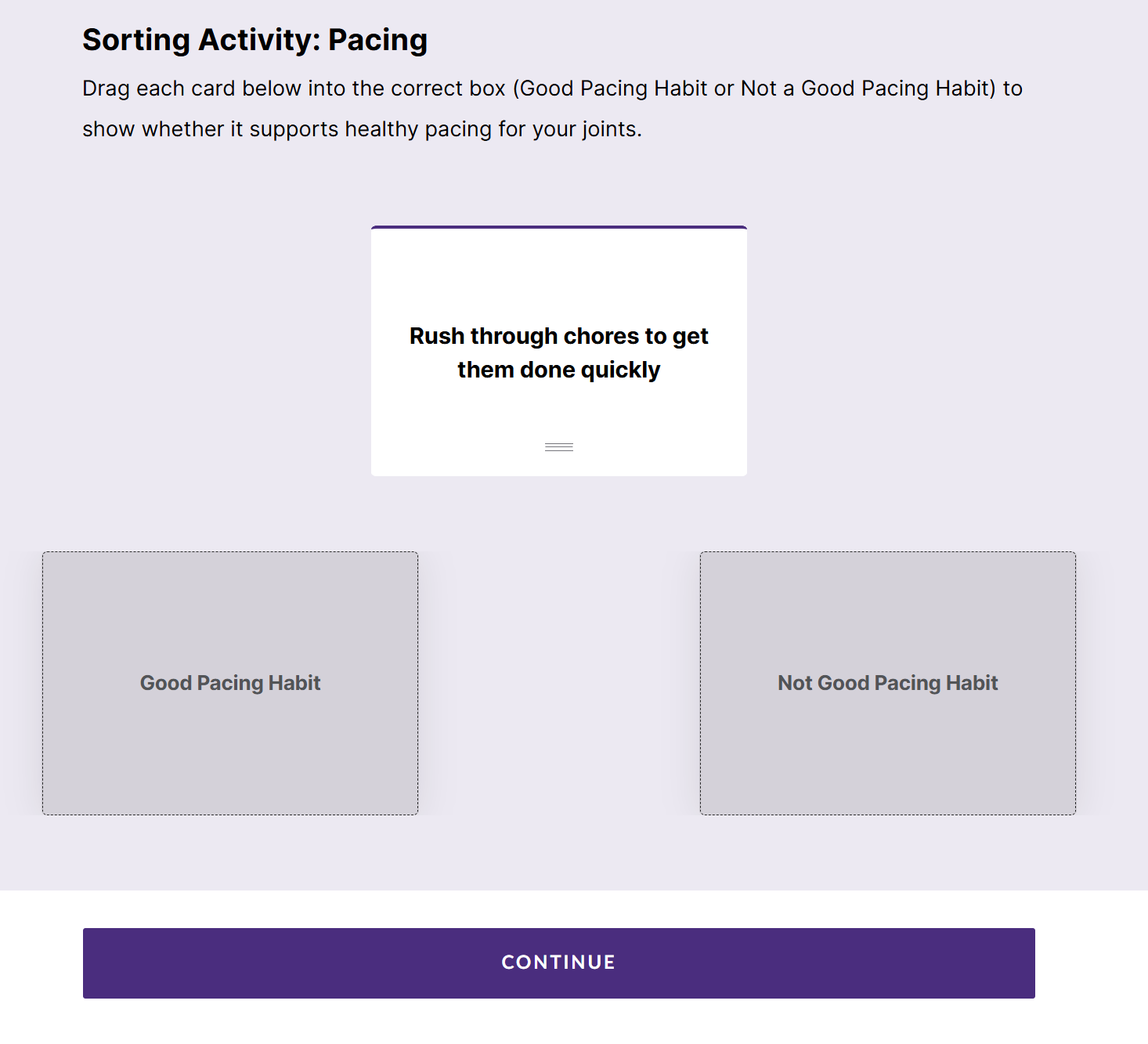
**

# **S5 Appendix. Flip card activity**


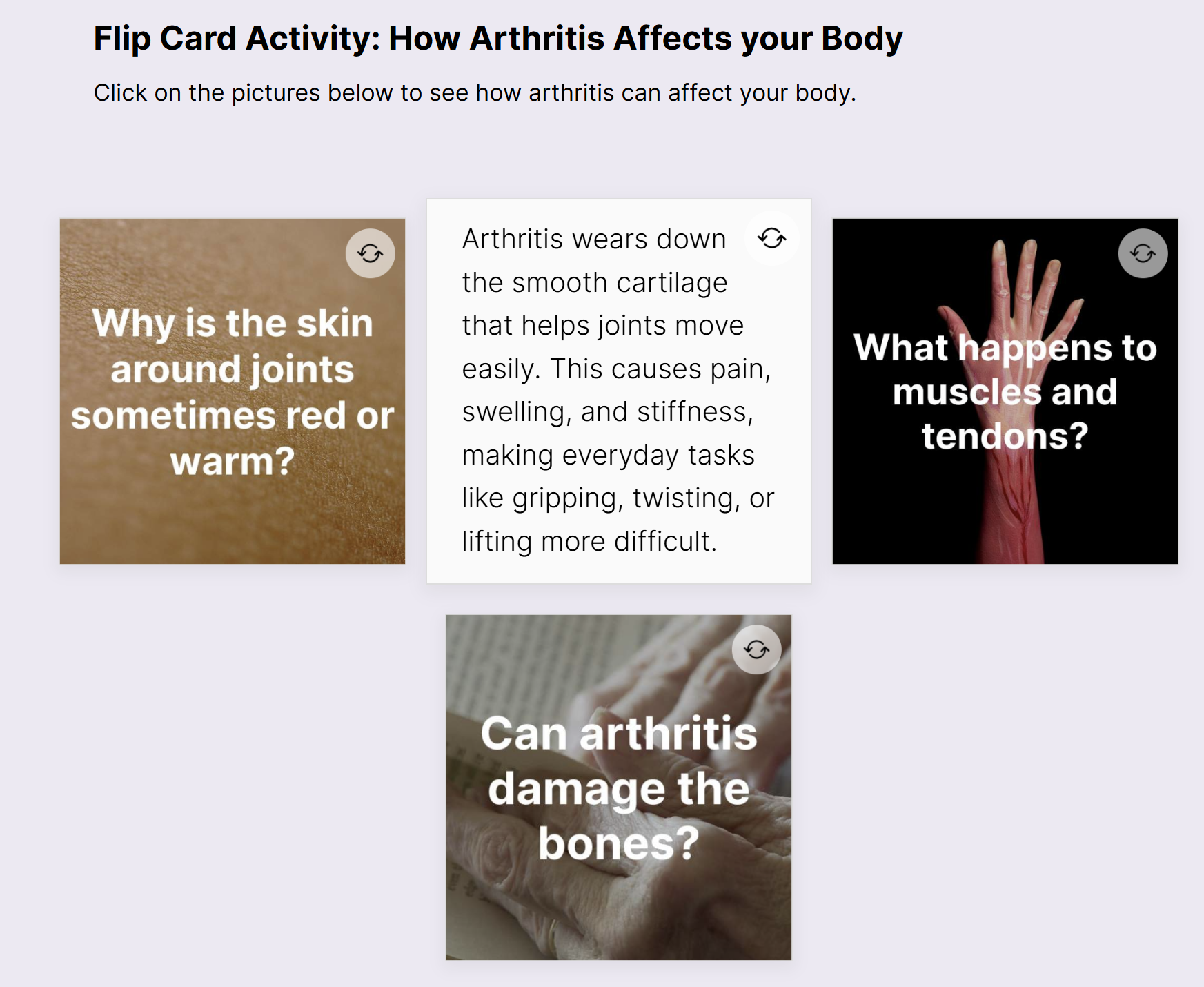


# **S6 Appendix. Quiz**

**
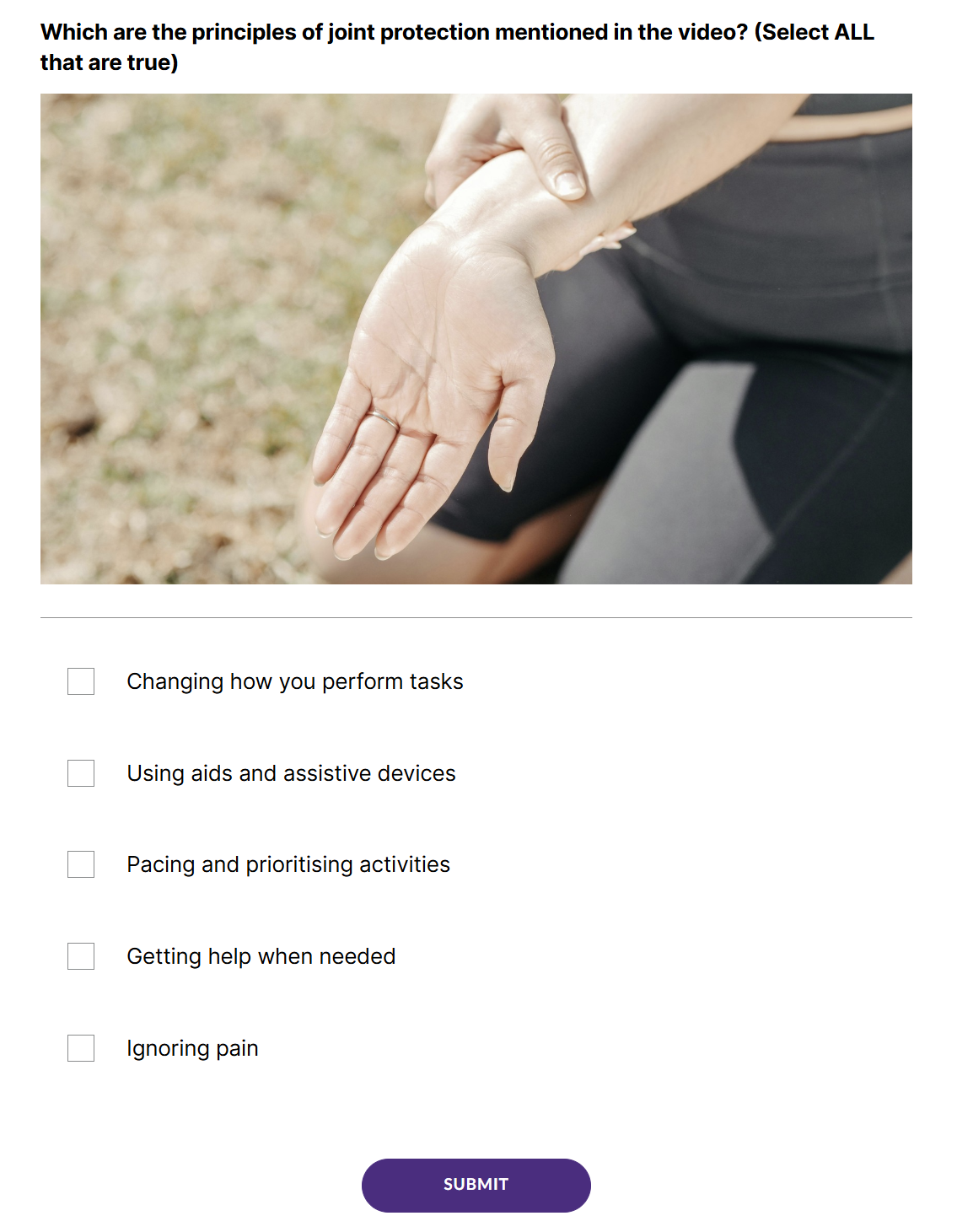
**

# **S7 Appendix. Interview Guides**

**Interview Guide – Session 1 (Free Navigation Session)**

**Purpose**
To test how intuitive the navigation of the Joint Protection Program is, how users interact with the program’s features and content, and to identify usability challenges.

**Duration**
30–60 minutes per participant.

**Objectives**

- Assess whether participants can complete standardized tasks successfully.
- Capture navigation errors and difficulties using eye-tracking.
- Observe how participants explore and interact with different types of content.

**Interviewer’s Role**

- Introduce and explain tasks.
- Ensure standardized task completion.
- Prompt participants to verbalize their thoughts (e.g., *“What are you thinking here?”* if they pause).

**Mode of Delivery**
Remote through Microsoft Teams, or in person at the HULC Clinical Research Lab (Room DB-222) using a designated laptop.

**Preamble**

“Hello, thank you for agreeing to participate in this research study. My name is Dimitra Pouliopoulou, and I am a PhD candidate at Western University in the Department of Health and Rehabilitation Sciences (Physical Therapy). In this study, we are testing a new remotely delivered Joint Protection Program to understand how intuitive it is to navigate and how users interact with its features.

During the session, I will first ask you to take a few minutes to get familiar with the platform. Then I will guide you through a set of tasks. Please verbalize your thoughts out loud while completing these activities. Since we are using eye-tracking, you will need to have your camera on. At the end, we will discuss your overall experience.

You will not be asked to provide personal information, and we kindly ask that you do not share personal details during the session. Do I have your permission to start recording?”

(*Wait for permission, answer questions, then begin recording.*)

**Part 1 – Familiarization and Standardized Tasks with Eye Tracking (~10 minutes)**

- 2–3 minutes: “Please take a moment to familiarize yourself with the platform.”
- Then complete standardized pre-defined tasks while eye-tracking records fixation points, scan paths, and skipped areas.

**Task List**
a. Locate the interactive house module.
b. Enter the “Kitchen” room.
c. Read the instructions on how to navigate the content.
d. Locate the “Opening a can” item.
e. Watch the video in full screen.
f. Exit full screen.
g. Return to the main house interface.
h. Skip to a different module.
i. Complete the drag-and-drop activity.
j. Locate the module *What is Hand Arthritis*.
k. Complete the flip-card activity.
l. Complete the scenario-based activity.

**Rating Prompt**
“On a scale from 0 to 10, how difficult was that, with 0 being very easy and 10 being very hard?”

**Part 2 – Free Exploration with Think-Aloud (~20–30 minutes)**

- “Now I’d like you to explore the program at your own pace. Please feel free to engage with quizzes, videos, drag-and-drop activities, or flip-card exercises.”
- “As you do this, please continue to verbalize your thoughts, impressions, and any challenges you encounter.”

**Prompt questions to use during exploration:**

- “What are you thinking here?”
- “Was that easy to find?”
- “What will you do next?”

**Post-Interaction Debrief (~20 minutes)**

1. How easy was it to navigate the program?
2. What features worked well?
3. What did you find confusing or frustrating?
4. Are there any features you think would be useful but are missing?
5. Is there anything else you would like to add?

**Interview Guide – Session 2 (Chunk-Specific Module Testing Session)**

**Purpose**
To assess usability and comprehension of specific program modules, focusing on clarity of language, ease of understanding, and confidence in applying the information.

**Duration**
30–60 minutes per participant.

**Objectives**

- Assess participants’ ability to complete standardized tasks successfully.
- Capture navigation errors and difficulties using eye-tracking.
- Evaluate clarity and comprehensibility of selected modules.

**Interviewer’s Role**

- Guide participants through steps as needed.
- Encourage think-aloud during module interaction.
- Collect ratings after each video.

**Mode of Delivery**
Remote through Microsoft Teams, or in person at the HULC Clinical Research Lab (Room DB-222).

**Preamble**

“Hello, thank you for agreeing to participate in this research study. My name is Dimitra Pouliopoulou, and I am a PhD candidate at Western University in the Department of Health and Rehabilitation Sciences (Physical Therapy). In this study, we are testing a new remotely delivered Joint Protection Program to understand how users interact with specific modules and how easy the information is to understand.

During the session, I will first ask you to take a few minutes to get familiar with the platform. Then I will guide you through a set of tasks. Afterward, you will review a selection of program videos and rate them on clarity and ease of understanding. Please verbalize your thoughts out loud as you go. Since we are using eye-tracking, you will need to have your camera on.

You will not be asked to provide personal information, and we kindly ask that you do not share personal details during the session. Do I have your permission to start recording?”

(*Wait for permission, answer questions, then begin recording.*)

**Part 1 – Familiarization and Standardized Tasks with Eye Tracking (~10 minutes)**

- 2–3 minutes: “Please take a moment to familiarize yourself with the platform.”
- Then complete standardized pre-defined tasks while eye-tracking records fixation points, scan paths, and skipped areas (same task set as in Session 1).

**Part 2 – Module-Specific Testing (~20–30 minutes)**

- Participants engage with:
  - Two pre-selected videos chosen by the research team to represent distinct content types.
  - Two additional videos of their own choice.
- While interacting, participants are instructed to think aloud (sharing impressions, challenges, and thought processes).

**After each video, participants are asked to rate:**

1. How clear was the language? Was the information to understand? (Rate 0-5)
2. Do you feel confident you could explain this information to someone else? (Rate 0-5)

**Final Debrief (~5–10 minutes)**

1. How did you feel about navigating these modules?
2. Were there any parts that felt confusing or unnecessary?
3. What would you change or improve?

# **S8 Appendix. Eye tracking coding**

Participant ID: P087

| Taks | Score (0-3) | Comment |
| --- | --- | --- |
| Locate Interactive House Module | 2 | Found it but took a second to figure out that the module tab needed to be clicked on to access the actual one   - *F Pattern but also Searching* |
| Enter a Room | 3 | Entered the kitchen immediately when prompted to   - *Reading with little searching* |
| Locate Instructions on how to interact with the content | 3 | Found and read, *reading pattern* evident |
| Locate an item inside the room | 2 | *Skipping/missing* when trying to find the can/jar opening |
| Watch the video related to the item | 3 | Completed no issues – *Reading* |
| Open a Video in Full Screen | 3 | Completed no issues – quick *Searching*, knew where it was |
| Exit Full Screen | 3 | Completed no issues - quick *Searching*, knew where it was |
| Return to main House interface | 3 | Located return button – after some *Searching* |
| Find and enter a different room | 3 | Completed no issues – quick *F Pattern* |
| Skip to a different module | 2 | Completed, knew modules were on the side but sometimes required *scanning* |
| Locate an interactive activity below a video | 3 | Always found them by scrolling down – *Reading and Z Pattern* |
| Complete a drag and drop shorting activity | 3 | Found and completed quickly. *Reading and Z Pattern* |
| Complete a quiz activity | 3 | Completed with little *Reviewing* |
| Complete a scenario-based activity | 3 | Completed with ease. *Reading* |
| Complete a flip card activity | 2 | Completed with *Reviewing* |

Participant ID: P047

| Taks | Score (0-3) | Comment |
| --- | --- | --- |
| Locate Interactive House Module | 3 | Located without issues. *F Pattern* while looking through the side bar |
| Enter a Room | 3 | Rooms located without issues. Smallest amount of *Scanning* to find the one prompted. |
| Locate Instructions on how to interact with the content | 3 | Found when prompted to - *Reading* |
| Locate an item inside the room | 2 | Could not find Jar very well – *Skipping/Missing and Searching* |
| Watch the video related to the item | 3 | Watched jar video when found - *Reading* |
| Open a Video in Full Screen | 3 | Completed no issues – quick *Searching* but found with ease |
| Exit Full Screen | 3 | Completed no issues - quick *Searching* but found with ease |
| Return to main House interface | 3 | Returned easily – super quick *searching* |
| Find and enter a different room | 3 | Found easily – *Z Pattern* over different rooms |
| Skip to a different module | 3 | Able to locate modules without instruction – *Z and F Pattern* through the side bar, *Reading* |
| Locate an interactive activity below a video | 3 | Always found them by scrolling – Smallest amount of *Scanning* |
| Complete a drag and drop shorting activity | 3 | No issues completing activity - *Reading* |
| Complete a quiz activity | 3 | Completed quiz, no issues – *Reading* |
| Complete a scenario-based activity | 2 | Some difficulty locating the activity but completed it without issues when found - *Searching* |
| Complete a flip card activity | 3 | Found on their own, completed without difficulty - *Reading and Z Pattern* |

Participant ID: P106

| Taks | Score (0-3) | Comment |
| --- | --- | --- |
| Locate Interactive House Module | 3 | Found without difficulty – *F Pattern* over all the room but did quickly |
| Enter a Room | 3 | Found the kitchen without problem |
| Locate Instructions on how to interact with the content | 0 | Not completed by user |
| Locate an item inside the room | 3 | Found the can without problem – quick *Scanning* |
| Watch the video related to the item | 2 | Found the video but had difficulty keeping the pop up open – *Problem Solving* and *Reviewing* |
| Open a Video in Full Screen | 3 | Completed – quick *Search* but just knew where it was |
| Exit Full Screen | 3 | Completed - quick *Search* but just knew where it was |
| Return to main House interface | 0 | Not completed by user |
| Find and enter a different room | 0 | Not completed by user |
| Skip to a different module | 3 | No issues finding other modules – *F and Z pattern* while looking through side bar |
| Locate an interactive activity below a video | 3 | Always found without difficulty – Small amount of *Scanning* |
| Complete a drag and drop shorting activity | 3 | Found and completed without difficulty - *Reading Z Pattern* while going through |
| Complete a quiz activity | 0 | Not completed by user |
| Complete a scenario-based activity | 3 | Found and completed without difficulty – *Reading Z Pattern* while going through |
| Complete a flip card activity | 3 | Found and completed without difficulty – *Reading and F Pattern* |

Participant ID: P045

| Taks | Score (0-3) | Comment |
| --- | --- | --- |
| Locate Interactive House Module | 3 | Located and opened without issues – *Reading* through sidebar |
| Enter a Room | 3 | Located and entered kitchen with ease – *Z Pattern* over the different rooms |
| Locate Instructions on how to interact with the content | 3 | Completed no issues – *Reading* through |
| Locate an item inside the room | 1 | A lot of difficulty finding the can (it seems like no one can find the can/jar) – *Skipping/Missing* and *Searching* |
| Watch the video related to the item | 3 | Watched can video - *Reading* |
| Open a Video in Full Screen | 0 | Platform error |
| Exit Full Screen | 0 | Platform error |
| Return to main House interface | 0 | Not completed by user |
| Find and enter a different room | 0 | Not completed by user |
| Skip to a different module | 3 | Found what is pacing module easily   - Small amount of *Searching,* knew it was in the sidebar |
| Locate an interactive activity below a video | 3 | Found without issues   - *Reading* through activity - Tiny amount of *reviewing* to make sure was doing right |
| Complete a drag and drop shorting activity | 2 | Completed but took a bit to understand what to do. Hard understanding some of the language   - *Problem Solving* and quick *reviewing* |
| Complete a quiz activity | 0 | Not completed by user |
| Complete a scenario-based activity | 0 | Not completed by user |
| Complete a flip card activity | 2 | Not automatically intuitive to user. Took a bit to get going   - *Problem Solving and Reviewing* |

Participant ID: P072

| Taks | Score (0-3) | Comment |
| --- | --- | --- |
| Locate Interactive House Module | 3 | Located and opened without issues – *Z Pattern* and *Reading* through the sidebar |
| Enter a Room | 3 | Located and entered kitchen with ease   - *F Pattern* and quick *scanning* while looking for prompted room |
| Locate Instructions on how to interact with the content | 3 | Found instructions without problems - *Reading* |
| Locate an item inside the room | 3 | Located multiple items without problems – quick *Z scanning* around |
| Watch the video related to the item | 3 | No issues – *Reading* during video |
| Open a Video in Full Screen | 3 | No Issues – quick *Searching* but knew where it was |
| Exit Full Screen | 3 | No issues - quick *Searching* but knew where it was |
| Return to main House interface | 3 | No issues – Quick *Searching* to find the button |
| Find and enter a different room | 0 | Not completed by user |
| Skip to a different module | 3 | Navigated through modules easily – *Reading* and little *scanning* |
| Locate an interactive activity below a video | 3 | Knew to scroll down and find activities   - *Reading* through activity instructions |
| Complete a drag and drop shorting activity | 3 | Understood how to complete. Some card phrasing was unclear – *Reading* with a little *Reviewing* |
| Complete a quiz activity | 0 | Not completed by user - *Skipping* |
| Complete a scenario-based activity | 2 | Completed but a lot of *scanning*, did not seem focused – slightly confused with task |
| Complete a flip card activity | 2 | Completed activity but *Reviewing* and some *Searching* evident |

Participant ID: P143

| Taks | Score (0-3) | Comment |
| --- | --- | --- |
| Locate Interactive House Module | 1 | Did not understand where the module was. Needed help to find it. Very prolonged S*earching* |
| Enter a Room | 3 | Located the kitchen without problems – small *Scanning* and then found |
| Locate Instructions on how to interact with the content | 3 | Required small S*canning* before locating the instructions in the corner. *Reading* through |
| Locate an item inside the room | 2 | Required some *scanning* and searching before locating the can |
| Watch the video related to the item | 2 | Uncertainty with how to get the video to play but got it eventually - *Reviewing* |
| Open a Video in Full Screen | Error | Platform Error |
| Exit Full Screen | Error | Platform Error |
| Return to main House interface | 1 | Skipping/Missing, Problem-solving |
| Find and enter a different room | 0 | Not completed by user |
| Skip to a different module | 3 | Able to locate What is Pacing module without any issues – *F Scanning* |
| Locate an interactive activity below a video | 3 | Found drag and drop activity without issues – quick *Searching, Reading* through instructions |
| Complete a drag and drop shorting activity | Error | Recording ended |
| Complete a quiz activity | 0 | Not completed by user |
| Complete a scenario-based activity | 0 | Not completed by user |
| Complete a flip card activity | 0 | Not completed by user |

Participant ID: P103

| Taks | Score (0-3) | Comment |
| --- | --- | --- |
| Locate Interactive House Module | 2 | *Scanning* and *searching* |
| Enter a Room | 3 | Located the kitchen without problems – *F Pattern* over the rooms and *Reading* room names |
| Locate Instructions on how to interact with the content | 3 | No issues finding instructions. Small *Searching* and then *Reading* through |
| Locate an item inside the room | 3 | Located the can quickly - *Searching* |
| Watch the video related to the item | 3 | Able to open without issues – small *searching* to find but found quickly |
| Open a Video in Full Screen | 0 | Platform Error |
| Exit Full Screen | 0 | Platform Error |
| Return to main House interface | 3 | Able to return without issues – quick *searching* to find |
| Find and enter a different room | 3 | Able to locate the garden/water plants pop up – required some *Scanning/ Z Scanning* |
| Skip to a different module | 3 | *Searching* and *Z scanning* |
| Locate an interactive activity below a video | 3 | Found drag and drop activity without issues – simply scrolled down, smallest amount of *Searching* |
| Complete a drag and drop shorting activity | 3 | Completed without issues – *Reading* instructions and cards |
| Complete a quiz activity | 0 | Not completed by user |
| Complete a scenario-based activity | 0 | Not completed by user |
| Complete a flip card activity | 0 | Not completed by user |

Participant ID: P021

| Taks | Score (0-3) | Comment |
| --- | --- | --- |
| Locate Interactive House Module | 3 | S*earching and Scanning* |
| Enter a Room | 3 | Found the kitchen without any issues – *Reading* room names, *Z Scanning* |
| Locate Instructions on how to interact with the content | 2 | *Reviewing* description and then realized there were in activity instructions - *Searching* |
| Locate an item inside the room | 1 | Struggled locating item, a lot of S*earching, Skipping/ Missing* |
| Watch the video related to the item | 0 | Not completed by user |
| Open a Video in Full Screen | 0 | Not completed by user |
| Exit Full Screen | 0 | Not completed by user |
| Return to main House interface | 0 | Not completed by user |
| Find and enter a different room | 0 | Not completed by user |
| Skip to a different module | 3 | Moved through multiple modules without issues – *Z Scanning* and *Reading* through side bar |
| Locate an interactive activity below a video | 3 | Found and completed without issues - *Reading* |
| Complete a drag and drop shorting activity | 0 | Not completed by user |
| Complete a quiz activity | 0 | Not completed by user |
| Complete a scenario-based activity | 2 | Completed but with a decent amount of *scanning* around |
| Complete a flip card activity | 0 | Not completed by user |
